# Combinatorial Effects of Protective Agents on Survival Rate of the Yeast Starter, *Saccharomyces cerevisiae* 88-4, after Freeze-Drying

**DOI:** 10.3390/microorganisms9030613

**Published:** 2021-03-16

**Authors:** Young-Wook Chin, Saerom Lee, Hwan Hee Yu, Seung Jae Yang, Tae-Wan Kim

**Affiliations:** Research Group of Traditional Food, Korea Food Research Institute, Iseo-myeon, Wanju-gun 55365, Jeollabuk-do, Korea; ywchin@kfri.re.kr (Y.-W.C.); zzz5624@naver.com (S.L.); Yuhwanhee@kfri.re.kr (H.H.Y.); yangseungjae@kfri.re.kr (S.J.Y.)

**Keywords:** yeast starter, freeze-drying, protectant, survival rate, response surface methodology

## Abstract

A yeast starter is formulated for commercial practices, including storage and distribution. The cell viability of the yeast starter is one of the most important factors for manufacturing alcoholic beverages to ensure their properties during the fermentation and formulation processes. In this study, 64 potential protective agents were evaluated to enhance the survival rate of the brewing yeast *Saccharomyces cerevisiae* 88-4 after freeze-drying. In addition, the optimized combination of protective agents was assessed for long-term storage. Finally, response surface methodology was applied to investigate the optimal concentration of each protectant. Twenty of the 64 additives led to an increase in the survival rate of freeze-dried *S. cerevisiae* 88-4. Among the various combinations of protectants, four had a survival rate >95%. The combination of skim milk, maltose, and maltitol exhibited the best survival rate of 61% after 42 weeks in refrigerated storage, and the composition of protectants optimized by response surface methodology was 6.5–10% skim milk, 1.8–4.5% maltose, and 16.5–18.2% maltitol. These results demonstrated that the combination of multiple protectants could alleviate damage to yeasts during freeze-drying and could be applied to the manufacturing starters for fermented foods.

## 1. Introduction

Yeast is one of the most important factors in determining the quality of alcoholic beverages because it produces ethanol as well as various flavor compounds, including alcohols, esters, and acids, during fermentation. Yeast starters are formulated as powders or slurries for commercial practices, including storage and distribution. Although numerous formulation technologies have been developed to date, freeze-drying is still primarily used for making microbial starters owing to its high cell survival rate over long periods [1]. In this process, the yeast is exposed to extreme environments, such as freezing and dehydration, which leads to cell death. In particular, the survival rate of the brewing yeast *Saccharomyces cerevisiae* after freeze-drying is considerably lower (<10%) than that of bacteria [2]. Thus, various additives such as sugars, polyols, polymers, proteins, antioxidants, amino acids, and natural substances have been assessed to improve the survival rate and fermentation performance after freeze-drying [3,4,5,6,7,8,9]. Some additives not only physically act as structural supporting materials and rehydration receptors via the replacement of water on macromolecules by amino or hydroxyl groups, but also biochemically protect cells from damage resulting from freezing and drying [4,10]. Depending on the type of protective agent, the protective effect could improve if two or more types are combined rather than adding a single one as the protection mechanism of each protectant is different [8]. Although some combinations of protective agents including skim milk, disaccharides (sucrose and trehalose), proteins (bovine serum albumin), and amino acids have been reported, they have been tested on lactobacilli for use as probiotics [11,12,13]. Concerning yeast, a few studies have reported the combinatorial effects of protective agents. It was reported that skim milk together with two additives, among trehalose, honey, sodium glutamate, or raffinose, increased the viability rate of *S. cerevisiae* cells from 30% to 96–98% after freeze-drying [4]. It was also reported that freeze-drying with a mixture of 1.8 kDa maltodextrin and trehalose improved the viability of *S. cerevisiae* CBS 1171 [5]. Abadias et al. showed that the survival of *Candida sake* cells increased from 0.2% to 30–40% by using a combination of skim milk and other sugars such as 5% or 10% lactose or glucose, and 10% fructose or sucrose [6]. Recently, Shu et al. optimized the concentrations of lactose, trehalose, and sodium glutamate as cryoprotectant composites using response surface methodology (RSM); thus, the survival rate of the probiotic yeast *Saccharomyces boulardii* improved to 64% [8]. Although some combinations of protective agents against freeze-drying have improved the survival rate of yeasts in the previous studies, these are less efficient in the industrial aspect because it contains relatively expensive sugars such as trehalose. Moreover, with regard to commercial practice, although the survival rate during long-term storage is much more important than the survival rate immediately after freeze-drying, there has been little research on the former.

The aim of this research was to develop a commercially feasible combination of protective agents for freeze-dried yeast starter used in the liquor industry. In our previous study, *S. cerevisiae* 88-4, isolated from a Korean fermentation starter, "*nuruk*" was screened as the best strain for manufacturing distilled spirits through various comparative analyses with industrial distillery yeasts. The *S. cerevisiae* 88-4 strain exhibited superior ability, not only in ethanol production but also in the formation of various aroma compounds, including ethyl caproate, ethyl caprylate, ethyl caprate, and phenylethyl acetate [14]. In this study, 64 potential protective agents were evaluated to enhance the survival rate of *S. cerevisiae* 88-4 after freeze-drying. In addition, the combinatorial effect of protective agents was investigated. The optimal combination of protective agents was assessed for long-term storage of 42 weeks. Finally, the concentrations of each protective agent were optimized to maximize survival rate using RSM via central composite design (CCD).

## 2. Materials and Methods

### 2.1. Yeast Strains and Inoculum Preparation

*S. cerevisiae* 88-4 (KCCM11456P), 98-4, 172-6, 192-4, H3-1, H4-1 and *Pichia anomalis* 161-7 isolated from *nuruk* were obtained from Korea Food Research Institute [14]. The yeast strains have been identified by sequencing of 26S rDNA or ITS region. The two industrial yeasts were acquired from liquor companies. The yeast strains were incubated at 30 °C for 18 h with shaking at 250 rpm in YPD (10 g/L yeast extract, 20 g/L peptone, and 20 g/L dextrose) (Difco, Detroit, MI, USA) broth, and cells were harvested to measure the survival rate. Cell suspensions were adjusted to approximately 10^7^ colony-forming units (CFU)/mL and used as cultures. It was stored at −80 °C in 15% glycerol solution until use in the experiments.

### 2.2. Addition of Protective Agents and Freeze-Drying Process

The protective agents (Table 1) were purchased from Sigma-Aldrich except d-mannose, d-xylose, d-fructose, d-galactose (Duchefa, Haarlem, The Netherlands), and glycerol, magnesium sulfate heptahydrate (Duksan, Ansan, Korea), and l-fucose (Carbosynth, Newbury, UK), and peptone, skim milk, yeast extract (Difco, Detroit, MI, USA), and taurine (Daejung, Siheung, Korea), and isomaltooligosaccharide, l-rhamnose (WAKO, Osaka, Japan), and d-maltose (YAKURI, Osaka, Japan).

To prepare the cells, *S. cerevisiae* 88-4 was cultured in YPD broth (1% inoculum) at 30 °C for 18 h, corresponding to approximately 10^7^ CFU/mL. Cells were centrifuged at 3000× *g* at 4 °C for 15 min and washed with 0.85% NaCl solution. Prior to the experiments, all protective agents were sterilized under UV for 18 h. The concentrations of each protectant were described in Table 1. Cells were resuspended in 1 mL protective agent solution as the freeze-dried sample, or 1 mL distilled water as a blank. Suspensions were frozen in a deep freezer at −80 °C for 3 h. Subsequently, freeze-drying was conducted under vacuum at 5 Pa for 24 h with a condenser temperature of −51 °C. Freeze-dried cells were immediately used to determine the survival rate. All protective agents were used directly with the yeast pellet.

### 2.3. Evaluation of Additives as a Protective Agent against Freeze-Drying

Sixty-four additives (Table 1) were tested to identify protective agents that could increase the survival rate of *S. cerevisiae* 88-4 after freeze-drying. The cell viability of freeze-dried *S. cerevisiae* 88-4 with the additives was indirectly evaluated by measuring the initial cell growth rate. Two milliliters of 64 additive solutions were added to cell pellets separated from cultures incubated for 18 h, followed by freezing for 3 h and freeze-drying for 40 h. To exclude the effect of additives on cell growth, freeze-dried *S. cerevisiae* 88-4 with additives was washed twice with double distilled water before incubation. Thereafter, 10 mL of YPD broth was added and incubated at 30 °C with shaking at 250 rpm. Following a 10 h incubation, the optical density at 600 nm was measured in a 96-well plate using a spectrophotometer. The relative cell growth rate was determined by subtracting the initial value of OD_600 nm_ of the culture from that of the OD_600 nm_ after 10 h based on the culture without additives.

### 2.4. Measurement of Survival Rate of Freeze-Dried Yeast Cells

After freeze-drying, each sample was rehydrated to original volume by adding distilled water. Thereafter, serially diluted samples were spread on YPD agar plates and incubated at 30 °C. After 24 h, colonies were counted. The survival rate was calculated as follows:

Survival rate (%) = the viable cell count number after freeze-drying (CFU/mL)/the viable cell count number before freeze-drying (CFU/mL) × 100(1)

Measurement of the survival rate was repeated independently in triplicate.

### 2.5. Measurement of Cell Growth Rate and Ethanol Production of Freeze-Dried Yeast Cells

To measure the cell growth rate and ethanol production of freeze-dried *S. cerevisiae* 88-4 cells, freeze-drying was performed as described above. Thereafter, freeze-dried *S. cerevisiae* 88-4 was incubated in 50 mL YPD medium at 25 °C with shaking at 80 rpm for 12 h. Cell growth was observed by measuring the OD_600 nm_. Concentrations of ethanol production were quantified using a high-performance liquid chromatography (HPLC) (Agilent 1200 series, Santa Clara, CA, USA) instrument equipped with a carbohydrate analysis column (Rezex ROA-Organic Acid, Phenomenex, Torrance, CA, USA). The culture medium was centrifuged and used for HPLC analysis after appropriate dilution. The column was heated at 60 °C, and 20 µL of the diluted supernatant was injected. Five millimoles of sulfuric acid solution was used as the mobile phase at a flow rate of 0.6 mL/min. Cell growth rate and ethanol production were measured independently in duplicate.

### 2.6. Optimization of Protective Agents Using Response Surface Methodology

Concentration optimization of each protectant was performed using RSM based on CCD. Skim milk, maltose, and maltitol were selected for CCD. The experimental number of this study was calculated according to the following equation:

N = k^2^ + 2k + C_p_ (2)

where k is the number of independent variables and C_p_ is the replicate number of the center point. The value of α was determined using the equation:

α = (2^k^)^1/4^ (3)

Supplementary Table S1 shows the codes and values of the three cryoprotectants at the five levels in the CCD. This study was conducted with three independent variables (percentage of skim milk, maltose, and maltitol) and five levels (−α, 1, 0, 1, α). The survival rate before and after freeze-drying was set as the dependent variable. This study was carried out in 18 experimental runs, as both k and Cp were 3. The α value of this design is 1.68. Protectant experiments for *S. cerevisiae* 88-4 were the same as those mentioned above. CCD design, data analysis, and RSM establishment were performed using the Design-Expert statistical software 12 (Stat-Ease Inc., Minneapolis, MN, USA).

### 2.7. Statistical Analysis

All experimental data were indicated as the mean ± standard deviation. One-way analysis of variance, two-way analysis of variance (for long-term storage experiment), and Duncan’s multiple range tests were used to determine significant differences among experimental results using SPSS version 20 (IBM Inc., Chicago, IL, USA).

## 3. Results

### 3.1. Evaluation of Additives as a Protective Agent against Freeze-Drying

As a preliminary experiment, the survival rate of seven yeast strains isolated from *nuruk* and two industrial distillery yeasts were measured after freeze-drying with 5% skim milk as a protectant. The survival rate of *S. cerevisiae* 88-4 was approximately 5.6%, which is the fourth highest among the seven strains isolated from *nuruk*; however, it was relatively low compared to that of industrial yeasts (Supplementary Figure S1). The survival rate of freeze-dried *S. cerevisiae* 88-4 without skim milk (88-4 con.) was only 0.01%.

**Figure 1:**
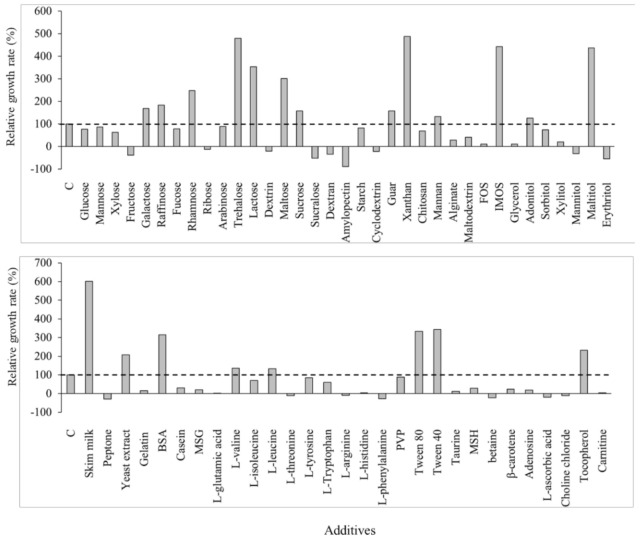
Relative cell growth rate of freeze-dried *Saccharomyces cerevisiae* 88-4 with various protectant agents. FOS, fructooligosaccharide; IMOS, isomaltooligosaccharide; BSA, bovine serum albumin; MSG, l-glutamic acid monosodium salt hydrate; PVP, polyvinyl pyrrolidone; MSH, magnesium sulfate heptahydrate.

Numerous protective agents have been assessed to enhance the survival rate of yeasts and lactic acid bacteria after freeze-drying. Based on previous studies, 64 additives were selected and evaluated as protective agents (Figure 1 and Table 1). The survival rates of *S. cerevisiae* 88-4 freeze-dried with additives were indirectly investigated by measuring the initial growth rate because it is proportional to the initial viable cell count. Among the 64 cultures, 20 cultures showed a higher cell growth rate than the control (freeze-dried cells without additives). In particular, skim milk (602%) showed the best protection effect, followed by xanthan gum (488%), d-trehalose (479%), isomaltooligosaccharide (443%), and maltitol (437%). Moreover, d-lactose (353%), Tween 40 (343%), Tween 80 (333%), bovine serum albumin (315%), d-maltose (301%), l-rhamnose (248%), tocopherol (232%), and yeast extracts (208%) also displayed a considerable protective effect against freeze-drying. d-Raffinose (183%), d-galactose (169%), sucrose (157%), guar gum (157%), adonitol (127%), l-valine (136%), and l-leucine (134%) exhibited marginal protective effects.

Skim milk has been widely used as a protectant against freeze-drying. Skim milk is a complex material composed of 52% lactose, 38% proteins, and trace elements; thus, it might have a better protective effect than other solitary substances [15]. Trehalose is also a well-known protectant in several organisms, including yeasts, bacteria, and plants [16]. It has been reported that trehalose protects cellular membranes from dehydration and increases thermal stability, thus it might act as a stabilizer of cellular structures under stressed conditions [17]. Despite the excellent protective effect of trehalose, its use as a cryoprotectant for yeast starter is limited owing to its relatively high price. The use of xanthan and guar gum appears unsuitable as a protective agent because of its high viscosity (data not shown). Maltitol also exhibited a considerable protective effect, as mentioned above. Oligosaccharide-derived sugar alcohols, such as maltitol, form a glass-state amorphous cake-structure, which protects proteins from activity loss caused by secondary structure perturbation during freeze-drying and storage [18]. Isomaltooligosaccharide is a type of prebiotic, and has been reported to display a protective effect in a test for the viability of lactic acid bacteria [19,20]. The nonionic surfactant Tween 80 has also been reported to have a protective effect on freeze-thawing of proteins such as lactate dehydrogenase and lysozyme by hampering its damaging interaction with ice crystals. The protective effect might be due to the competition between Tween molecules and the protein for sites on the ice surface [21,22,23]. Although the protective mechanism remains unclear, some hypotheses including water replacement, preferential exclusion, hydration force explanation, and vitrification of sugars have described the mechanism [10].

### 3.2. Effect of Skim Milk Concentration on Survival Rate of Freeze-Dried Yeast Cells

**Figure 2:**
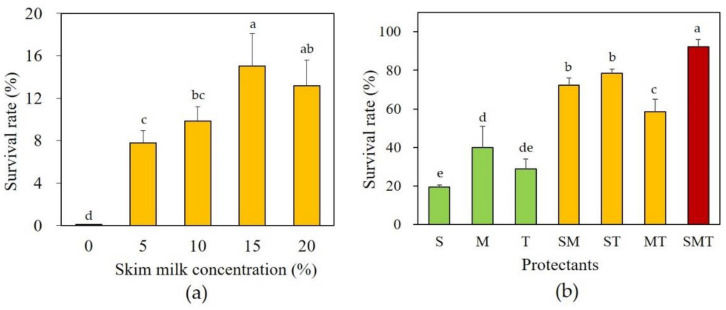
(**a**) Effect of skim milk concentration and (**b**) combinatorial effects of selected protectants on the survival rate of *S. cerevisiae* 88-4 after freeze drying. S, skim milk (15%); M, maltose (10%); T, trehalose (10%). ^a–e^ Values with different letters on the bar indicate significant differences (*p* < 0.05).

As mentioned above, among the 64 additives, skim milk showed the best protective effect against freeze-drying. To determine the optimal concentration of skim milk, freeze-drying at a concentration of 0% to 20% was performed, and the survival rate was measured (Figure 2a). As a result, the survival rate of freeze-dried *S. cerevisiae* 88-4 without skim milk was extremely low (0.27%). An improvement in the survival rate was observed when the concentration of skim milk was increased from 0% to 15% (Figure 2a). However, the survival rate did not significantly increase at concentrations above 15% (*p* > 0.05). Therefore, 15% was determined to be the optimal concentration of skim milk. It was confirmed that there is a limitation in improving the survival rate with a single protective agent, and it could be improved further in combination with other protective agents because the protective mechanism may work differently for each agent. Therefore, subsequent experiments were conducted to increase the survival rate of *S. cerevisiae* 88-4 by combining several protective agents.

### 3.3. Combinatorial Effect of Protectants on Survival Rate of Freeze-Dried Yeast Cells

To test the combinatorial effect of protective agents, three well-known protectants, skim milk (15%), maltose (10%), and trehalose (10%) were selected. As shown in Figure 2b, the survival rates were found to be between 20% and 40% when a single protective agent was added. For double combination protectants, survival rates increased to 59–78.5%. Triple combination protectants resulted in a significantly higher survival rate (95%, *p* < 0.05) than that of the single or double-protectants. The combination of skim milk, trehalose, and maltose considerably enhanced the survival rate after freeze-drying. The improved protective effect of combination protectants was also confirmed by other protective agents (Supplementary Figure S2).

### 3.4. Investigation of Optimal Combination of Protectants against Freeze-Drying

**Figure 3:**
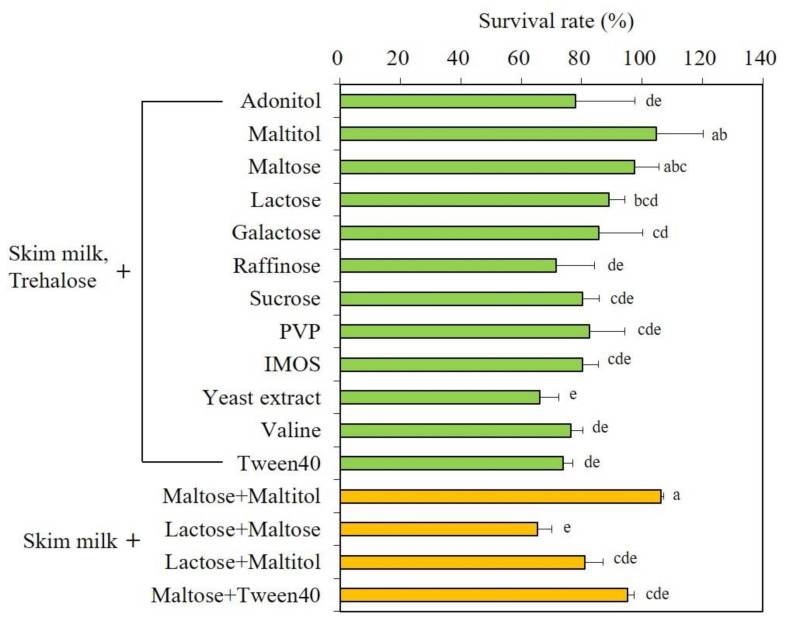
Triple combinatorial effects of various protectants on the survival rate of *S. cerevisiae* 88-4 after freeze-drying. PVP, polyvinyl pyrrolidone; IMOS, isomaltooligosaccharides. ^a–e^ Values with different letters on the bar indicate significant differences (*p* < 0.05).

To investigate the optimal combination of protectants, the survival rate of freeze-dried *S. cerevisiae* 88-4 with various combinations of protectants was measured. As skim milk and trehalose exhibited the best protective effect (Figure 1), these two protective agents were fixed, and the other protective agent was added as a variable to the test. As shown in Figure 3, maltitol (105%) displayed the highest survival rate, followed by maltose (95%) and lactose (89%). The survival rate of other protective agents was almost similar or decreased compared to the case of adding only skim milk and trehalose. As trehalose is a relatively expensive sugar, the survival rate was also investigated with cheaper protectants (Figure 3 bottom). The combination of skim milk, maltose, and maltitol showed the highest survival rate, which was similar to that of skim milk, trehalose, and maltitol. The combination of skim milk, maltose, and maltitol was not significantly different from that of skim milk, trehalose, and maltitol (*p* > 0.05). As mentioned above, the cell growth rate depends on the initial number of viable cells. To investigate the effects of protectant combinations on cell growth of freeze-dried *S. cerevisiae* 88-4, cell growth was compared in flask cultures (Supplementary Figure S3). The control (freeze-dried without protectant) did not grow until 12 h of incubation. In contrast, the combination of skim milk, maltose, and maltitol showed the highest cell growth rate. The cell growth rate was displayed in survival rate order in the four combinations of protective agents.

### 3.5. Stability of Freeze-Dried Yeast Cells in Long-Term Storage

**Figure 4:**
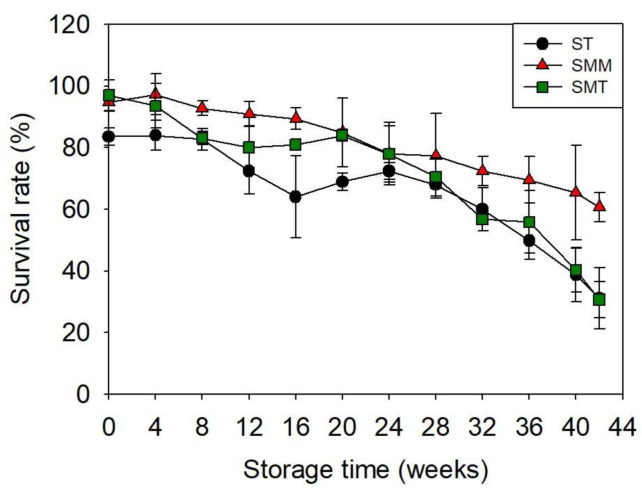
Changes in the survival rate of freeze-dried *S. cerevisiae* 88-4 during long-term storage under refrigerated conditions. ST, skim milk/trehalose; SMM, skim milk/maltose/maltitol; SMT, skim milk/maltose/tween 40.

Two cost-effective protectant combinations, skim milk/maltose/maltitol (SMM) and skim milk/maltose/Tween 40 (SMT), which showed high protective effects, were selected to investigate the long-term stability of freeze-dried *S. cerevisiae* 88-4. The combination of skim milk and trehalose (ST) was used as a control. The freeze-dried *S. cerevisiae* 88-4 with protectant combinations was stored at 4 °C for 42 weeks, and the survival rate was measured intermittently (Figure 4 and Table 2). Immediately after the freeze-drying process, the combination of SMT and SMM showed survival rates of 97% and 95%, respectively, which were >10% higher than that of ST. As the storage period increased, the survival rate gradually decreased in all three samples (*p* < 0.05). Following six months of storage, the survival rate was maintained above 70% in all samples. However, the survival rate rapidly decreased in ST and SMT samples. At 42 weeks of storage, the survival rate of SMM was 61%, which was twofold higher than that of ST and SMT. Therefore, the combination of SMM was demonstrated most suitable for long-term refrigerated storage of *S. cerevisiae* 88-4.

### 3.6. ANOVA Results of Central Composite Design and Response Surface Model

The combination of skim milk, maltose, and maltitol, which is a cost-effective protectant with excellent protective effects against freeze-drying and long-term refrigerated storage ability, was selected for CCD. The CCD of the protective effect of a protectant for *S. cerevisiae* 88-4 is shown in Table 3. The protectant combinations at the set concentration showed a survival rate of 22.73% to 95.60% after freeze-drying. According to the CCD results, the multiple regression equation is as follows:

Y = 84.58 − 6.30A − 8.97B − 2.84C − 0.95AB + 0.2AC − 14.93BC − 15.23A^2^ − 9.31B^2^ − 1.44C^2^ (4)

In the equation, Y is the survival rate of freeze-dried *S. cerevisiae* 88-4, and A, B, and C represent skim milk, maltose, and maltitol, respectively.

The analysis of variance for the regression analysis is presented in Table 4. The *p* values of the response surface model showed the significance of the model (*p* < 0.01), while the values of lack of fit were not significant (*p* > 0.05), which indicated that the model was significant, and the regression analysis was available for modeling RSM. As the multiple regression equation was suitable to reflect actual experimental data, it could analyze the result of Y according to the selected independent variable. An *R*^2^ = 0.9047 indicated that the operated response surface of 90.47% could be explained by this model, further indicating that the experimental data and regression equation were well fitted. In addition, *p* values of the primary terms A and B, the interactive term B*C, and the quadratic terms A^2^ and B^2^ were below 0.05, indicating that they had a significant influence on the designed model.

**Figure 5:**
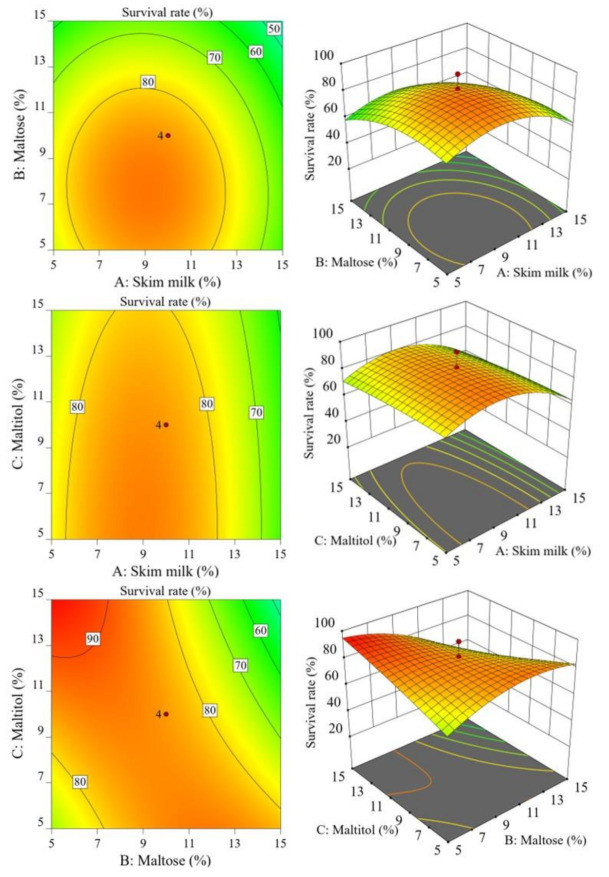
The contour plots and response surface models for the protective effect of selected protectants on the survival rate of *S. cerevisiae* 88-4 after freeze drying. A, Skim milk (%), B, Maltose (%), and C, Maltitol (%).

The survival rate of freeze-dried *S. cerevisiae* 88-4 (Y) and the protective effect of selected factors containing skim milk (A), maltose (B), and maltitol (C) were designed using contour plots and 3D response surface models, as shown in Figure 5. The formulation of skim milk, maltose, and maltitol as protectants was optimized to attain the maximum survival rate after freeze-drying using the multiple regression equation. The optimized protectant formulation was determined according to Derringer’s desirability function. The appropriate protectant formulation corresponded to concentrations of 6.5–10% skim milk, 1.8–4.5% maltose, and 16.5–18.2% maltitol to obtain a maximum survival rate of *S. cerevisiae* 88-4 after freeze-drying. As a verification experiment, the optimized protectant consisting of skim milk (9%), maltose (4.5%), and maltitol (18%) was tested to improve the survival rate of *S. cerevisiae* 88-4 after freeze-drying. The survival rate of *S. cerevisiae* 88-4 after freeze-drying was 94.2% (data not shown). RSM using CCD was statistically suitable for optimizing the concentration of protectants composed of skim milk, maltose, and maltitol. It could efficiently increase the survival rate of *S. cerevisiae* 88-4 after freeze-drying.

## 4. Discussion

Freeze-drying with protectants to ensure optimal microbial viability plays an important role in food industry and microbiology. The survival rate of freeze-dried microorganisms is influenced by several factors including protectants, freezing temperature, cooling rate and dehydration method. This study was focused on the composition of protectants because they are relatively easy to apply to industrial process and the impact is extremely large. Among the 64 additives evaluated, 20 enhanced the survival rate of freeze-dried *S. cerevisiae* 88-4. The most effective protectant group was saccharides, in particular, disaccharides including trehalose, lactose, maltose and sucrose (Figure 1). In terms of protection mechanisms, the balance among vitrification, interactions between sugars and proteins and global and local mobility of protein is crucial in preserving the protein. Smaller sugars generally are more suitable for molecular interactions (i.e., hydrogen bond) and reduction of local mobility [24], whereas larger sugars (i.e., oligosaccharides) are generally more appropriate for vitrification. Therefore, relatively small sugar (i.e., disaccharides) such as trehalose might be ideal to stabilize the proteins under freeze-drying condition [25]. Trehalose has been used in protectant combination in many previous studies because of its excellent protective effect [4,5,8,26,27]. The performance of trehalose was also confirmed in this study. Nevertheless, trehalose is still expensive compared to other sugars to use as a protectant for microorganism starters.

Combined protectants generally displayed better protection performance than singular ones. This might be because each protectant works differently; thus, the combination may lead to synergistic effects. The combinatorial protective effect fairly depended on the type of protectant (Figure 2 and Figure 3). Some combinations of protective agents (skim milk, trehalose, sucrose, raffinose, and lactose) tested in this study have also been confirmed in previous studies [4,6,8]. A new cost-effective combination of protectants composed of skim milk, maltose and maltitol was found in this study. The combination showed that it can effectively protect yeast not only immediately after freeze-drying but also during long-term storage. Notably, in RSM using CCD, which was conducted to optimize the composition of the protectants, it was found that the protective effect increased when more maltitol was added rather than skim milk or maltose. Thus, maltitol may play a key role in the combinatorial protective effect. Maltitol has been reported to be used as a protective agent against freeze-drying of proteins [18], gold nanoparticles [28], and kiwiberry [29], but it has not yet been applied for yeasts. Moreover, maltitol has been reported as a potential protectant owing to its relatively high glass transition temperature (−34.1 °C) along with amorphous solid state after freeze-drying [30]. Further research will be conducted to maximize the stability during long-term storage and cost efficiency by combining more types of protective agents while lowering their concentration.

## 5. Conclusions

Protective agents are one of the most critical factors determining the survival rate and fermentation performance of freeze-dried yeast starters. In this study, a wide variety of protective agents were assessed to improve the survival rate of *S. cerevisiae* 88-4 after freeze-drying. Among the 64 additives, 20 were found to enhance the survival rate of *S. cerevisiae* 88-4. Although skim milk showed the best protective effects against freeze-drying, its protective effect as a single additive was limited. Combination of protectants led to a considerable improvement in the survival rate. Among the various protectant combinations, four exhibited survival rates >95% immediately after freeze-drying. In particular, the combination of 15% skim milk, 10% maltose, and 10% maltitol maintained a survival rate of 80% during the six months of refrigerated storage. In the optimization of protectant composition through RSM using CCD, a combination of skim milk (6.5–10%), maltose (1.8–4.5%), and maltitol (16.5–18.2%) maximized the survival rate of *S. cerevisiae* 88-4 after freeze-drying. The enhanced survival rate could be attributed to the fact that the three kinds of additives protect yeast cells with different mechanisms thus, exhibiting a synergistic effect. The optimized composition of protectants can ensure improved quality of microbial starters and hence can be applied to various fields including manufacturing microbial starters for fermented food industry, production of probiotics, preservation of microbial strain bank, and fecal microbiota transplantation.

## Figures and Tables

**Table 1:** List of protective agents and its concentration used in this study.

Protectantive Agents	Conc. (%)	Protectantive Agents	Conc. (%)	Protectantive Agents	Conc. (%)
**Monosaccharides**		Isomaltooligosaccharide (IMOS)	1	l-Isoleucine	1
l-Arabinose	10	Maltodextrin	5	l-Leucine	1
d-Fructose	10	Mannan	0.5	Sodium glutamate (MSG)	10
l-Fucose	10	d-Raffinose	10	l-Phenylalanine	1
d-Galactose	10	Starch	10	l-Threonine	1
d-Glucose	10	Xanthan gum	10	l-Tryptophan	1
d-Mannose	10	**Sugar alcohols**		l-Tyrosine	1
l-Rhamnose	10	Adonitol	10	l-Valine	1
d-Ribose	10	Erythritol	10	**ETC**	
d-Xylose	10	Glycerol	10	Adenosine	0.1
**Disaccharides**		Maltitol	10	l-Ascorbic acid	2.5
d-Lactose	10	d-Mannitol	10	Betaine	15
d-Maltose	10	d-Sorbitol	10	β-Carotene	1
Sucrose	10	Xylitol	10	l-Carnitine	2.5
d-Trehalose	10	**Proteins**		Choline chloride	1
**Oligosaccharides**		Bovine serum albumin (BSA)	10	Magnesium sulfate heptahydrate (MSH)	0.75
Alginate	2	Casein	10	Polyvinylpyrrolidone (PVP)	5
Amylopectin	10	Gelatin	3	Skim milk	10
Chitosan	0.5	Peptone	10	Sucralose	10
β-Cyclodextrin	5	Yeast extract	4	Taurine	7.5
Dextran	10	**Amino acids**		d-Tocopherol	0.3
Dextrin	10	l-Arginine	1	Tween 80	1
Fructooligosaccharide (FOS)	1	l-Glutamic acid	1	Tween 40	1
Guar gum	10	l-Histidine	1		

**Table 2:** Effect of various protectant compositions on survival rate of freeze-dried *S. cerevisiae* 88-4 during long-term storage under refrigerated condition.

Weeks	Combinations of Protectants		
	ST	SMM	SMT
0	83.56 ± 2.78 ^Ab^	94.70 ± 2.86 ^Aa^	96.97 ± 4.92 ^Aa^
4	84.00 ± 4.81 ^Aa^	97.35 ± 6.56 ^Aa^	93.51 ± 7.23 ^ABa^
8	82.67 ± 3.53 ^Ab^	92.80 ± 2.37 ^ABa^	83.12 ± 2.25 ^BCb^
12	72.44 ± 2.04 ^Bb^	90.91 ± 3.94 ^ABa^	80.09 ± 6.66 ^CDb^
16	64.00 ± 1.33 ^BCDc^	89.39 ± 3.47 ^ABa^	80.95 ± 0.75 ^CDb^
20	68.89 ± 2.78 ^BCb^	84.85 ± 11.15 ^ABCa^	83.98 ± 1.98 ^BCa^
24	72.44 ± 2.78 ^Ba^	78.00 ± 10.20 ^BCDa^	77.92 ± 9.09 ^CDa^
28	68.0 ± 1.33 ^BCa^	77.27 ± 13.78 ^BCDa^	70.50 ± 6.14 ^Da^
32	60.0 ± 7.06 ^CDb^	72.35 ± 4.73 ^CDEa^	56.71 ± 3.75 ^Eb^
36	63.11 ± 6.16 ^BCDa^	69.50 ± 7.57 ^CDEa^	55.84 ± 10.14 ^Ea^
40	55.11 ± 8.68 ^Da^	65.40 ± 15.30 ^DEa^	40.26 ± 7.23 ^Fa^
42	31.11 ± 10.01 ^Eb^	60.70 ± 4.80 ^Ea^	30.67 ± 5.77 ^Fb^

All values are means ± standard deviations of three replicates. ^A–F^ and ^a–c^ in the same column and row are significantly different (*p* < 0.05, Duncan’s multiple range tests), respectively. ST, skim milk/trehalose; SMM, skim milk/maltose/maltitol; SMT, skim milk/maltose/tween 40.

**Table 3:** Central composite design for the composite protectant of *S. cerevisiae* 88-4.

Run	Skim Milk (A, %)	Maltose (B, %)	Maltitol (C, %)	Survival Rate (%)
1	5	15	5	84.00
2	10	10	10	95.60
3	10	10	10	81.20
4	10	10	10	84.62
5	10	10	18.4	86.96
6	5	15	15	31.25
7	15	15	5	57.14
8	10	10	10	76.32
9	15	15	15	22.73
10	15	5	5	55.17
11	5	5	15	85.20
12	5	5	5	60.71
13	15	5	15	62.97
14	18.4	10	10	36.36
15	1.6	10	10	50.00
16	10	1.6	10	75.86
17	10	18.4	10	44.00
18	10	10	1.6	77.41

**Table 4:** The ANOVA results for the experimental model.

Source	Sum of Squares	df	Mean Square	F-Value	*p*-Value	Significant
Model	7082.08	9	786.90	8.44	0.0031	**
A	542.82	1	542.82	5.82	0.0423	*
B	1098.84	1	1098.84	11.78	0.0089	**
C	110.44	1	110.44	1.18	0.3082	
A*B	7.22	1	7.22	0.0774	0.7879	
A*C	0.3362	1	0.3362	0.0036	0.9536	
B*C	1783.24	1	1783.24	19.12	0.0024	**
A2	2935.81	1	2935.81	31.48	0.0005	***
B2	1097.00	1	1097.00	11.76	0.0090	**
C2	26.32	1	26.32	0.2823	0.6096	
Residual	745.97	8	93.25			
Lack of fit	544.96	5	108.99	1.63	0.3655	
Pure error	201.01	3	67.00			
R2	0.9047					
Adjust R2	0.7975					

A, skim milk; B, maltose; C, maltitol; *** *p* < 0.001; ** *p* < 0.01; * *p* < 0.05.

## Data Availability

This article did not report any data.

## Supplementary Materials

The following are available online at https://www.mdpi.com/2076-2607/9/3/613/s1, Figure S1: Survival rate of seven yeasts isolated from nuruk and two industrial distillery yeasts after freeze-drying, Figure S2: Comparison of protective effects of various protectant combinations on survival rate of S. cerevisiae 88-4 after freeze-drying, Figure S3: Effects of protectant combinations on cell growth (a) and ethanol production (b) of freeze-dried S. cerevisiae 88-4, Table S1: The levels of the protective agents in the CCD.
