# Supplementary material for: Combinatorial Effects of Protective Agents on Survival Rate of the Yeast Starter, Saccharomyces cerevisiae 88-4, after Freeze-Drying
Source: Microorganisms. 2021 Mar 16;9(3):613. doi: 10.3390/microorganisms9030613 (PMC8002499; doi:10.3390/microorganisms9030613)
Supplement: Supplementary file 1 [file microorganisms-09-00613-s001.pdf]

Supplementary Tables and Figures

Table S1. The levels of the protective agents in the CCD

Factor	Symbol	Level of factors				
		$-\alpha$	-1	0[a]	1	α
Skim milk (%)	A	1.6	5	10	15	18.4
Maltose (%)	B	1.6	5	10	15	18.4
Maltitol (%)	C	1.6	5	10	15	18.4

a, Central point

α, 1.68

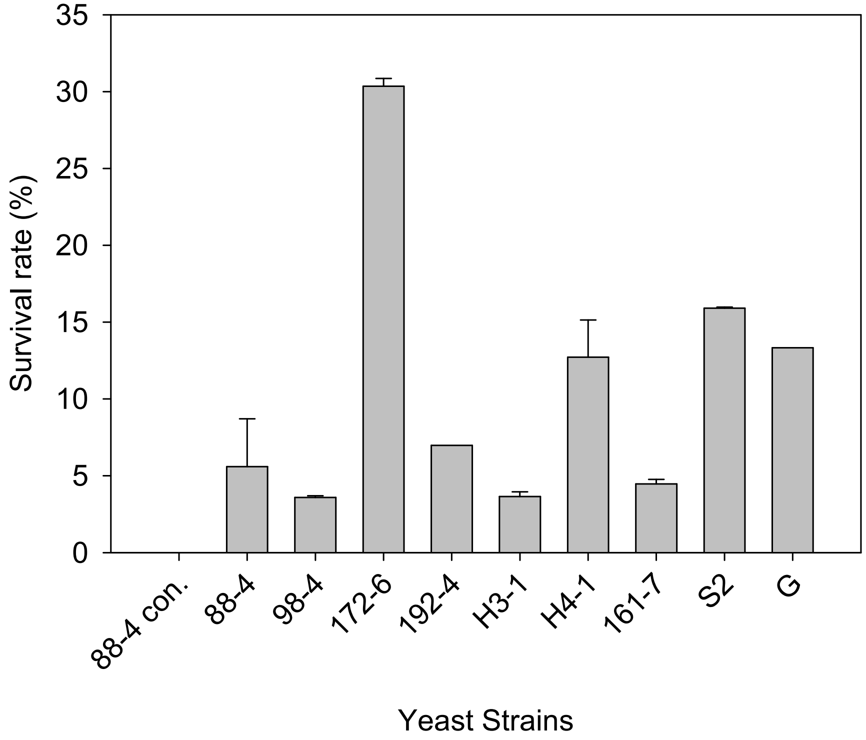

Figure S1. Survival rate of seven yeasts isolated from *nuruk* and two industrial distillery yeasts after freeze-drying

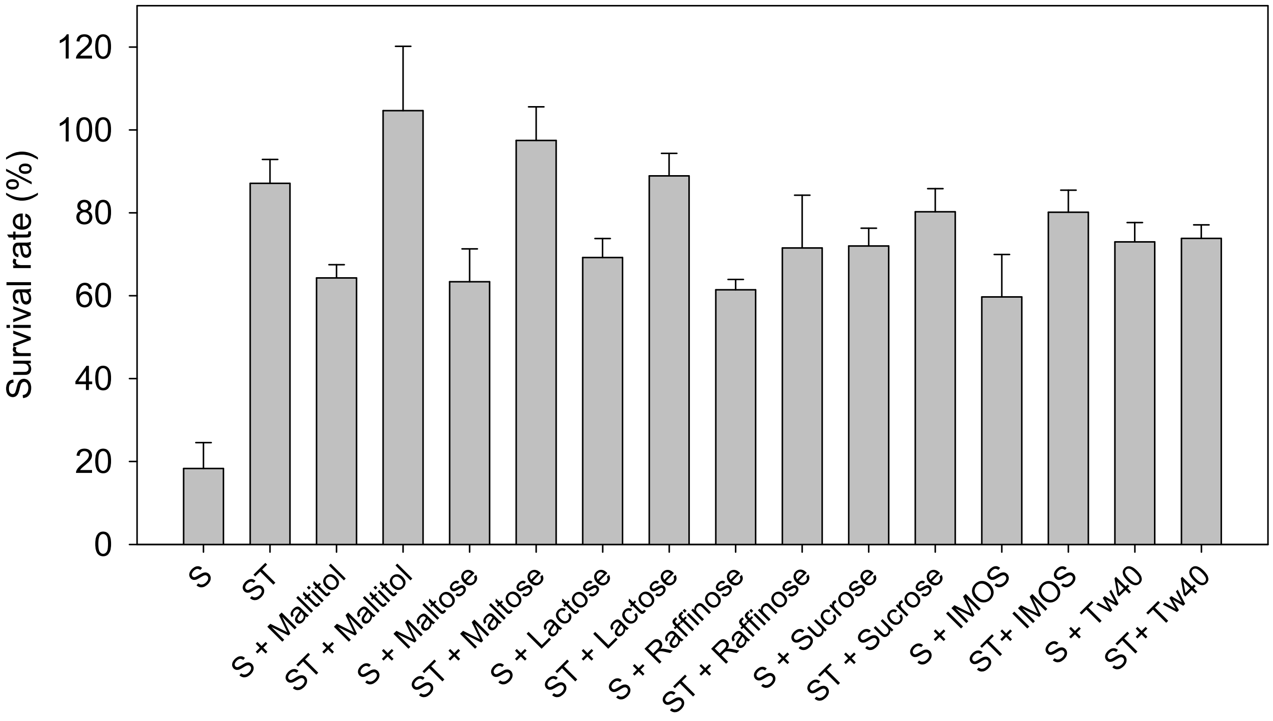

Figure S2. Comparison of protective effects of various protectant combinations on survival rate of *S. cerevisiae* 88-4 after freeze-drying; S, skim milk (15%); ST, skim milk (15%) / trehalose (10%); IMOS, isomaltooligosaccharide (1%); Tw40, Tween 40 (1%). Concentration of the protectants is listed in Table S1.

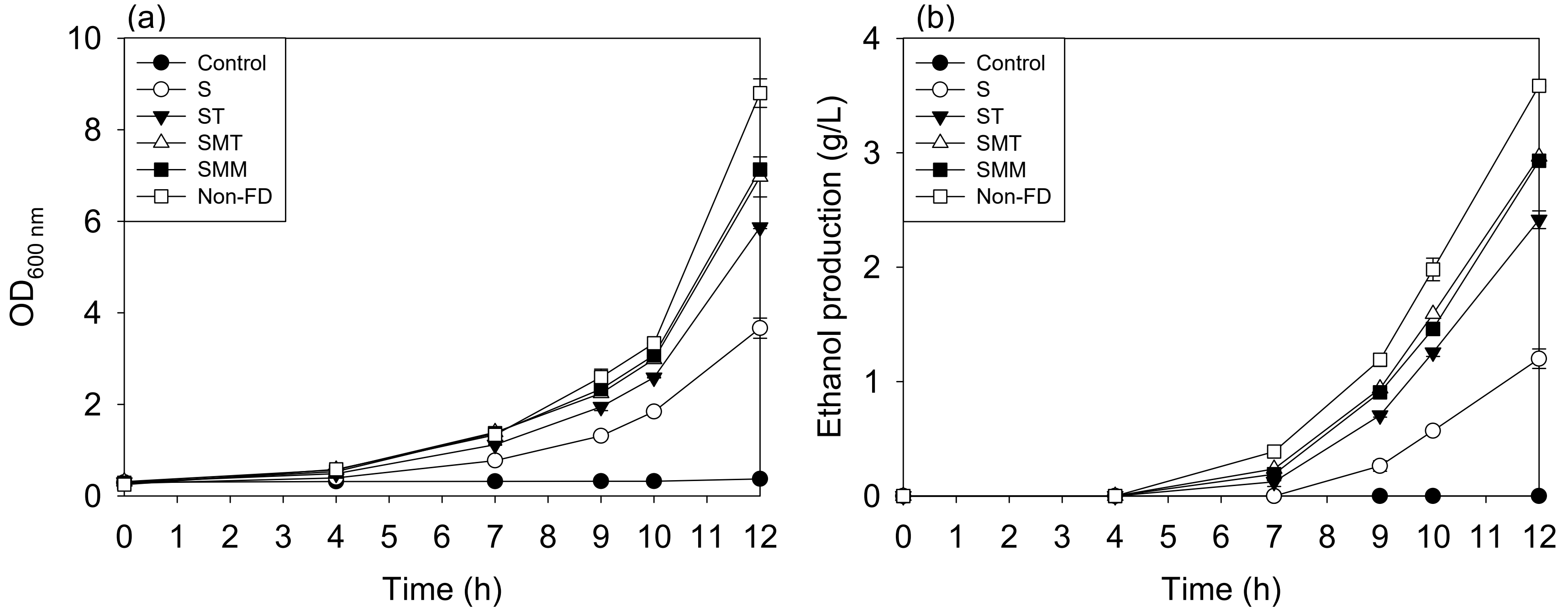

Figure S3. Effects of protectant combinations on cell growth (a) and ethanol production (b) of freeze-dried *S. cerevisiae* 88-4. Control, freeze-dried without protectant; S, skim milk; ST, skim milk/trehalose; SMT, skim milk/maltose/tween 40; SMM, skim milk/maltose/maltitol; Non-FD, not freeze-dried *S. cerevisiae* 88-4. Concentration of the protectants is listed in Table S1.
